# Antenatal and Postnatal Maternal Depressive Symptoms and Trajectories and Child Hospitalization up to 24 Months of Life: Findings From the 2015 Pelotas (Brazil) Birth Cohort Study

**DOI:** 10.1016/j.ympdx.2021.100065

**Published:** 2021

**Authors:** Nadège Jacques, Marilia Arndt Mesenburg, Joseph Murray, Andréa Dâmaso Bertoldi, Marlos Rodrigues Domingues, Alan Stein, Mariangela Freitas Silveira

**Affiliations:** 1 Postgraduate Program in Epidemiology, Federal University of Pelotas, Pelotas, Brazil; 2 Federal University of Health Sciences of Porto Alegre, Porto Alegre, RS, Brazil; 3 College of Physical Education, Federal University of Pelotas, Pelotas, Brazil; 4 Department of Psychiatry, University of Oxford and MRC/Wits Rural Public Health and Health Transitions Research Unit (Agincourt), School of Public Health, Faculty of Health Sciences, University of the Witwatersrand, Johannesburg, South Africa

**Keywords:** EPDS, Edinburgh Postnatal Depression Scale

## Abstract

**Objective:**

To examine the association between antenatal and postnatal maternal depression symptoms, and child hospitalization during the first 2 years of life in the 2015 Pelotas Birth Cohort Study.

**Study design:**

This is an observational study. Maternal depressive symptoms of 4275 mothers were measured using the Edinburgh Postnatal Depression Scale. Hospitalization of the child for any reason was assessed using maternal report. Bivariate analysis and multivariate Poisson regressions were used to assess the association between maternal depressive symptoms and child hospitalization.

**Results:**

Compared with children of mothers with low depressive symptoms, children whose mothers experienced significant antenatal depressive symptoms were 1.74 (95% CI, 1.16-2.60) times more likely to be hospitalized by 3 months of age, and 2.14 (95% CI, 1.46-3.14) times more likely up to 24 months. For children whose mothers experienced severe postnatal depressive symptoms at 3 months, the risks for hospitalization by age 12 months were 1.84 (95% CI, 1.39-2.45) higher than children whose mothers had low depressive symptoms. There was an increased risk of hospitalization for children according to the severity of depressive trajectories across time.

**Conclusions:**

Maternal depressive symptoms are a risk factor for hospitalization in children up to 2 years of age, and this risk increases with increased severity of depression. These results have public health relevance for decreasing the risk factors in mothers that can lead to hospitalization in children.

Child health is fundamental to future and lifelong wellbeing. While children are rapidly developing, many environmental factors influence health and disease, potentially resulting in health service use.^1^, ^2^, ^3^, ^4^, ^5^ Maternal depression arises from a combination of biological and psychosocial factors, and decades of studies have shown that both antenatal and postnatal maternal depression are associated with adverse effects on both mothers and children.^6^, ^7^, ^8^, ^9^, ^10^, ^11^, ^12^, ^13^ In this context, maternal depression may be a risk factor for child ill health and hospitalization.^14^, ^15^, ^16^ Hospitalization is a stressful experience for both parents and children; in addition to being a high-risk environment for a young child, it represents a high cost that could be decreased through preventive strategies to promote child health.^17^, ^18^, ^19^

A few studies evaluated the effect of maternal depression on child physical health or hospitalization, many from high-income countries.^20^, ^21^, ^22^, ^23^, ^24^, ^25^, ^26^, ^27^ Few examined antenatal maternal depression, and those that started antenatally examined child outcomes only in the first days postnatally.^21^^,^^23^ Thus, there is a need to study this relationship longitudinally, which should allow us to see how maternal depressive symptoms may influence the physical health of children and to study this relationship in the context of upper-middle-income, low, and low-middle-income countries (World Bank category) because of the differences in the social and economic factors. Our aim was to study the association between antenatal and postnatal maternal depressive symptoms and depressive symptoms trajectories, on child hospitalization during the first 2 years of life, among children from the 2015 Pelotas Birth Cohort Study, Brazil.

## Methods

This study was carried out using data from the 2015 Pelotas Birth Cohort Study, a longitudinal study of all live births in Pelotas, a midsized city in southern Brazil.^28^ Unlike previous cohort studies conducted in the same city, the 2015 cohort recruited mothers during pregnancy; all mothers expected to deliver in 2015 were identified through an active search strategy. For the antenatal period, the main assessment questionnaire was completed at 20 weeks of gestation (range, 16-24 weeks of gestation). For the perinatal follow-up visit, interviews were performed 24-48 hours after delivery. For the 3- and 12-month follow-ups, mothers were interviewed at home, and for the 24-month follow-up, interviews were performed at the research center. More information is available in the 2015 Cohort profile.^28^

### Measures

#### Maternal Depressive Symptoms

Maternal depressive symptoms were measured antenatally at 20 weeks gestational age (range, 16-24 weeks), and at the 3-, 12-, and 24-month postnatal visits using the Edinburgh Postnatal Depression Scale (EPDS).^29^, ^30^, ^31^, ^32^ During all follow-up visits, the EPDS was administered by trained interviewers.

The EPDS is composed of 10 items, each coded from 0 to 3.^29^ It was validated in the 2004 Pelotas Birth Cohort Study for use in Pelotas, Brazil, with the best cutoff point for screening postnatal depression being 10 or higher, with 82.6% (range, 75.3%-89.9%) sensitivity and 65.4% (range, 59.8%-71.1%) specificity.^33^ For the purpose of this analysis, maternal depressive symptoms were categorized as 0-9, low; 10-12, moderate; 13 or more, significant; and 15 or more, severe. We treated the EPDS as a continuous variable for the trajectory group analysis.

#### Child Hospitalization

Hospitalization of a child for any reason during the years 2015 and 2016 was based on the following questions: “Has the child been hospitalized since birth until now (3 months), at 3 months until now (12 months) and at 12 months until now (24 months)?” (yes/no); “How old was the child when he/she was hospitalized?” (for each time); and “What was the reason for each hospitalization?”

#### Covariates

The following information on mothers and children was collected during the perinatal interview. Maternal age in years (grouped as <20, 20-34, and ≥35); maternal skin color (White, Black/mixed); maternal educational attainment (grouped into 0-4 years, no schooling/incomplete junior high school; 5-8 years, junior high school/incomplete high school; 9-11 years, high school/incomplete higher education; and 12 years or more, higher education degree); Brazilian economic classification criterion (ABEP), based on purchasing power, measured by the ownership of household assets (treated as a categorical variable in this analysis: A-B, upper and upper middle social class; C, lower middle social class; D-E, lower social class); marital status (lives with partner, yes/no); having pregestational depression (based on this question: “Before this pregnancy have you experienced depression?” yes/no); smoking during pregnancy (regular smokers were defined as those who smoked at least one cigarette daily in any trimester of pregnancy; yes/no); consumption of alcohol during pregnancy (any amount of alcohol intake during any trimester of pregnancy was considered as alcohol consumption during pregnancy; yes/no); parity (number of previous deliveries, including stillbirths, grouped as primiparous, 2 children; 3 children and more); planned pregnancy, yes/no; threatened miscarriage (yes/no); preeclampsia/eclampsia (yes/no); heart disease during pregnancy (yes/no); gestational diabetes (yes/no); and gestational hypertension (yes/no). Child variables were recorded at birth: gestational age (18 to <37, preterm; 37 to ≥ 42, term); sex (male or female); birth weight (<2500 g, low; ≥2500 g, normal); 5-minute Apgar score (≤ 6 or ≥7); breastfeeding (exclusive, predominantly, partial, weaned). For maternal smoking and alcohol intake, we assumed that a mother who smokes and drinks alcohol regularly during pregnancy would smoke and drink after childbirth.

### Statistical Analyses

Mother-child pairs were described according to maternal depressive symptoms measured antenatally, and at the 3- and 12-month assessments. A bivariate analysis was performed using Poisson regression and we considered only covariates who shown an association with the outcomes to be included in the multivariate analysis for each period. A multivariate Poisson regression analysis was performed to assess the association between maternal depressive symptoms during the antenatal and postnatal (3 and 12 months) periods and children's hospitalization at 3, 12, and 24 months, while controlling for the covariates cited elsewhere in this article.

The maternal depressive symptoms trajectory for the subjects who completed the EPDS on at least 3 follow-up visits (including, necessarily, the antenatal enrollment visit) was estimated through group-based trajectory modeling. This semiparametric method proposed by Nagin and Tramblay^34^ and Tramblay et al^35^ aims to identify groups of individuals that manifest a similar trajectory by evaluating the outcome of interest on at least 3 time points. Missing data were retained in the analysis and handled by the model through maximum likelihood estimation. A polynomial function model was used, assuming a censored normal (c norm) distribution, designed for analysis of approximately continuous scales, measured repeatedly, that can be censored by a minimum or maximum scale or both.^36^ The model facilitates disclosure of the relationship between maternal EPDS score, gestational age, and child age, using age for time indexing.^34^^,^^35^^,^^37^ The number and shape of the trajectories were chosen based not only on best model fit, evaluated through the maximum Bayesian information criterion, but also on the interpretability of the trajectories obtained.^35^ Selection of the appropriate model was guided by posterior probability scores for each trajectory group (ie, the individual's probability of belonging to each of the trajectory groups). Those with a mean probability score of greater than 0.7 were selected for all groups according to Nagin and Tremblay.^35^

Multivariable Poisson regression analyses were performed to assess the association between child hospitalization for the whole period (3, 12, and 24 months) with each trajectory group. Models were estimated using the “traj” command in Stata.^36^

For all analyses, we considered results significant where a *P* value of less than < .05, and report 95% CIs. All analyses were performed in Stata version 12.0 (StataCorp LP, 2011. Stata Statistical Software: Release 12).

## Results

All 4387 children, born in hospitals in the urban area of Pelotas (>99% of births), between January 1 and December 31, 2015, were eligible for inclusion in the cohort study. Of these, 4275 mothers (98.7%) agreed to participate. The response rates at the follow-up visits were 4110 (97.2%) at 3 months, 4018 (95.4%) at 12 months, and 4216 (95%) at 24 months. Of all 4275 mothers who participated in the perinatal assessment, 3155 (73.8%) were interviewed during pregnancy.^28^

Table I describes sample characteristics according to proportion of depressive symptoms during the antenatal period and at 3 and 12 months after birth. Maternal depressive symptoms across all time points (antenatal and 3 months and 12 months postnatal) were significantly associated with each other. During pregnancy, mothers’ own reports of having been depressed before pregnancy were also associated with high levels of depressive symptoms measured on the EDPS at each timepoint in the study.

**Table I:** Maternal and child characteristics by maternal antenatal and postnatal depressive symptoms: 2015 Pelotas Birth Cohort, Brazil

Maternal characteristics	Depressive symptoms		
	Antenatal (n = 3132)	3 Months postnatal (n = 4095)	12 Months postnatal (n = 3972)
Maternal age, years	<.001	.010	<.001
<20	211 (38.5)	187 (24.3)	253 (34.3)
20-34	592 (28.1)	525 (19.3)	734 (27.7)
≥35	122 (25.6)	119 (19.9)	151 (25.9)
Skin color	<.001	<.001	<.001
Black/mixed	336 (39.9)	309 (26.5)	380 (33.8)
Schooling, years	<.001	<.001	<.001
0-4	128 (56.6)	148 (40,7)	149 (43.7)
5-8	324 (44.7)	275 (26.2)	394 (39.1)
9-11	313 (27.5)	257 (18.2)	366 (26.3)
≥12	160 (15.4)	151 (11.9)	229 (18.6)
ABEP (economic level)	<.001	<.001	<.001
A-B	160 (16.4)	165 (13.6)	230 (19.4)
C	487 (31.0)	393 (19,9)	564 (29.2)
D-E	248 (50.2)	237 (30.8)	303 (41.6)
Marital status	<.001	<.001	<.001
Without partner	178 (43.9)	186 (32.0)	205 (37.6)
Depression before pregnancy	<.001	<.001	<.001
Yes	287 (53.5)	183 (34.1)	222 (42.4)
Smoking during pregnancy	<.001	<.001	<.001
Yes	221 (50.5)	221 (33.5)	269 (42.7)
Alcohol during pregnancy	.015	<.001	<.001
Yes	78 (36.9)	97 (32.2)	112 (38.5)
Parity	<.001	<.001	<.001
Primiparous	368 (22.9)	306 (15.1)	471 (23.9)
2 children	301 (30.7)	275 (21.6)	350 (28.2)
≥3 children	255 (46.5)	250 (31.5)	317 (41.6)
Planned pregnancy	<.001	<.001	<.001
No	541 (35.2)	514 (24.2)	684 (33.4)
Threat of miscarriage	<.001	.008	<.001
Yes	110 (40.3)	89 (25.9)	123 (36.8)
Preeclampsia/eclampsia	.184	.147	.044
Yes	71 (33.6)	61 (23.8)	87 (34.2)
Heart disease	.006	.240	.091
Yes	23 (48,9)	15 (26.8)	21 (39.6)
Antenatal depressive symptoms	<.001	<.001	
Yes	_	382 (42.3)	475 (53.9)
3 Months postnatal depressive symptoms		<.001	
Yes	_	_	509 (64.8)
Gestational age, weeks	.739	.169	.370
Preterm (18 to <37)	138 (30.2)	134 (22.4)	174 (30.2)
Sex		.816	1.000
Male	_	423 (20.4)	579 (28.6)
Female	_	408 (20.1)	560 (28.7)
Birthweight	.791	.551	.630
<2500 g	87 (28.7)	83 (21.4)	110 (29.7)
APGAR at 5 minutes	.708	.833	.346
≤6	11 (32.3)	6 (17.1)	7 (20.6)
Breastfeeding	<.001	<.001	<.001
Exclusive	321 (23.1)	292 (15.9)	438 (24.6)
Predominant	86 (37.5)	71 (23.3)	98 (33.2)
Partial	274 (35.3)	253 (25.3)	316 (32.9)
Weaned	226 (33.4)	215 (22.6)	279 (31.2)

Fisher exact test was considered for all analysis.

Values are number (%) and *P* values.

A higher proportion of depressive symptoms at the antenatal period and 3 and 12 months afterwards were found among mothers younger than 20 years, with lower schooling and socioeconomic levels, Black or mixed skin color, living alone, with 3 or more children, having had an unplanned pregnancy, having depressive symptoms before pregnancy, who smoked or consumed alcohol during pregnancy, and having a threatened miscarriage.

### Antenatal and Postnatal Symptoms of Depression

The proportion of women who had antenatal depressive symptoms was 29.5% (95% CI, 27.9-31.1) of which 13.4% (95% CI, 12.2-14.6) had moderate depressive symptoms, 7.6% (95% CI, 6.7-8.5) had significant depressive symptoms, and 8.5% (95% CI, 7.5-9.5) experienced severe depressive symptoms. At 3 months postnatal, 20.2% of mothers (95% CI, 19.0-21.5) experienced depressive symptoms of which 9.2% (95% CI, 8.3-10.1) had moderate, 5.6% (95% CI, 4.9-6.3) significant, and 5.4% (95% CI, 4.7-6.1) severe depressive symptoms. The proportion of mothers who experienced depressive symptoms at 12 months postnatal was 28.7% (95% CI, 27.3-30.1) of which, 12.6% (95% CI, 11.6-13.6) had moderate symptoms, 8.1% (95% CI, 7.2-8.9) had significant symptoms, and 8.0% (95% CI, 7.2-8.9) had severe depressive symptoms.

### Antenatal and Postnatal Depression Symptoms and Child Hospitalization

Between birth and 3 months of age, 7.3% of children (95% CI, 6.5-8.1) were hospitalized, 16.8% (95% CI, 15.6-17.9) were hospitalized between 3 and 12 months of age, and 7.8% (95% CI, 7.0-8.7) were hospitalized between 12 and 24 months of age.

Table II shows the relative risk of hospitalization for children born to mothers who experienced antenatal depressive symptoms, categorized as low, moderate, significant, or severe. Compared with children of mothers who experienced low depressive symptoms, a child whose mother experienced antenatal significant depressive symptoms had the highest risk of hospitalization: 1.74 times (95% CI, 1.16-2.60) the risk of hospitalization by 3 months of age; 1.36 times (95% CI, 1.05-1.77) the risk of hospitalization at 12 months; and 2.14 times (95% CI, 1.46-3.14) the risk of hospitalization at 24 months. For children whose mothers experienced severe depressive symptoms at 24 months of age, the risk of hospitalization was 1.87 (95% CI, 1.25-2.81) compared with children of mothers who experienced low depressive symptoms at the antenatal period.

**Table II:** Child's hospitalization in accord with antenatal maternal depressive symptoms, 2015 Pelotas Birth cohort, Brazil

Maternal depressive symptoms (n = 3132)	3 Months hospitalization		12 Months hospitalization		24 Months hospitalization	
	Crude	Adjusted	Crude	Adjusted	Crude	Adjusted
Antenatal depression symptoms	<.001	.044	<.001	.092	<.001	.004
Low	1.00	1.00	1.00	1.00	1.00	1.00
Moderate	1.51 (1.05-2.17)	1.12 (0.75-1.66)	1.29 (1.02-1.63)	1.01 (0.79-1.29)	1.49 (1.05-2.13)	1.28 (0.90-1.83)
Significant	2.28 (1.56-3.31)	1.74 (1.16-2.60)	1.78 (1.39-2.29)	1.36 (1.05-1.77)	2.53 (1.79-3.59)	2.14 (1.46-3.14)
Severe	1.56 (1.02-2.39)	1.00 (0.61-1.62)	1.81 (1.43-2.30)	1.22 (0.94-1.60)	2.39 (1.70-3.37)	1.87 (1.25-2.81)

Values are relative risk and (95% CI).

Adjusted for maternal age, skin color, schooling, income, marital status, depression before pregnancy, planned pregnancy, smoking and alcohol during pregnancy, parity, threat of miscarriage, preeclampsia/eclampsia, and heart disease.

Table III shows the relative risk of hospitalization by children of mothers who experienced postnatal depressive symptoms. Compared with children of mothers who experienced low depressive symptoms at 3 months after birth, the risk of hospitalization at 12 months of age was 1.84 times higher (95% CI, 1.39-2.45) for children whose mothers experienced severe depressive symptoms at the same time.

**Table III:** Child's hospitalization in accord with 3 and 12 months postnatal maternal depressive symptoms, 2015 Pelotas Birth Cohort, Brazil

Maternal depressive symptoms	12 Months hospitalization		24 Months hospitalization	
	Crude	Adjusted	Crude	Adjusted
3 Months postnatal depression symptoms (n = 4095)	<.001	.004	<.001	.053
Low	1.00	1.00	1.00	1.00
Moderate	1.47 (1.19-1.82)	1.17 (0.88-1.54)	1.64 (1.18-2.27)	1.27 (0.83-1.93)
Significant	1.54 (1.18-1.99)	1.16 (0.81-1.64)	2.61 (1.90-3.59)	1.89 (1.19-2.99)
Severe	2.39 (1.95-2.93)	1.84 (1.39-2.45)	2.09 (1.45-3.01)	1.44 (0.84-2.47)
12 Months postnatal depression symptoms (n = 3972)			<.001	.045
Low			1.00	1.00
Moderate			1.45 (1.05-2.11)	1.24 (0.85-1.83)
Significant			1.98 (1.42-2.77)	1.66 (1.09-2.53)
Severe			2.85 (2.14-3.80)	1.72 (1.10-2.67)

The 3 months postnatal depression is adjusted for maternal age, skin color, schooling, income, marital status, depression before pregnancy, planned pregnancy, smoking and alcohol during pregnancy, parity, threat of abortion, preeclampsia/eclampsia, heart disease, antenatal depressive symptoms, gestational age, sex, birthweight, 5-minute APGAR, and breast-feeding.

The 12 months postnatal depression is adjusted for maternal age, skin color, schooling, income, marital status, depression before pregnancy, planned pregnancy, smoking and alcohol during pregnancy, parity, threat of abortion, preeclampsia/eclampsia, heart disease, antenatal depressive symptoms, gestational age, sex, birthweight, 5-minute APGAR, breast-feeding, and 3 months postnatal depression symptoms.

Compared with children of mothers who experienced low depressive symptoms at 3 months after birth, the risk for hospitalization at 24 months, was 1.89 times higher (95% CI, 1.19-2.99) for children whose mothers experienced significant depressive symptoms at the same time. Compared with children of mothers who experienced low depressive symptoms at 12 months after birth, the risk for hospitalization at 24 months was 1.66 times higher (95% CI, 1.09-2.53) for children whose mothers experienced significant depressive symptoms at the same time. Finally, compared with children of mothers who experienced low depressive symptoms at 12 months after birth, the risk for hospitalization at 24 months was 1.72 times higher (95% CI, 1.10-2.67) when mothers experienced severe depressive symptoms at the same time.

One of the main reasons for hospitalization during the first year of life was lower respiratory tract diseases (48% of total hospitalizations in this period); the mothers of one-third of children hospitalized with this illness had depressive symptoms at 3 months postnatal (Table IV).

**Table IV:** Main reasons for hospitalization during the first year old, 2015 Pelotas Birth Cohort, Brazil

Diseases	n = 675 (%)	Antenatal depressive symptoms (n = 3132) (%)	3 Months postnatal depressive symptoms (n = 4095)
		*P* < .001	*P* < .001
Diseases of external ear			
Otitis, earache/irritation	15 (2.2)	5 (45,4)	5 (33.3)
Allergic or hypersensitivity conditions			
Asthma, bronchospasm, shortness of breath	52 (7.7)	22 (63.8)	20 (39.2)
Gastroenteritis or colitis of infectious origin			
Food allergy	13 (1.9)	8 (72.7)	5 (38.4)
Urinary tract infection, site not specified			
Urinary infection	25 (3.7)	5 (26.3)	6 (26.1)
Unspecified jaundice			
Jaundice, light bath, yellowish	36 (5.3)	5 (16.7)	4 (11.1)
Surgery admission	19 (2.8)	3 (17.6)	4 (21.1)
Respiratory instability of prematurity			
Hyaline membrane disease	21 (3.1)	2 (15.4)	4 (19.1)
Fever of other or unknown origin			
Fever	18 (2.6)	7 (53.8)	6 (33.3)
Febrile seizures unspecified			
Convulsion	11 (1.6)	50 (50.0)	5 (45.4)
Infectious gastroenteritis or colitis without specification of infectious agent			
Diarrhea, intestinal infection	8 (1.2)	3 (60.0)	3 (42.8)
Upper respiratory tract disorders	27 (4.0)	9 (52.9)	12 (44.4)
Cold, flu, stuffy nose,	5		
Cough	1		
Tonsillitis, Laryngitis	4		
Respiratory infection	17		
Lower respiratory tract diseases	321 (48)	94 (40.5)	100 (31.6)
Bronchitis and bronchiolitis	226		
Bronchopneumonia pneumonia	95		
Others	103 (15.3)	27 (34.2)	29 (26.6)

Values are number (%).

### Trajectory Groups from Antenatal to 24 Months Postnatal Depressive Symptoms and Hospitalization in Children

All 3040 mothers (78%) who completed the EPDS during the antenatal period and in at least 2 other follow-up visits were included in the present analysis. We identified 5 trajectory groups of maternal depression. The first (low depressive symptoms) considered as no symptoms for this study and the second (moderate low depressive symptoms) in the threshold of the cutoff, groups had an EPDS score of less than 10 at all-time points and included 76.6% of the mothers. The third group (increasing depressive symptoms) included 9.8% of the mothers, who exhibited a constant increase in EPDS scores over the period of analysis. The fourth group (decreasing but persistent depressive symptoms) included 9.7% of the mothers, who presented a high depressive symptom score in the antenatal period, had a decrease at 3 months, and maintained a score around 10 between the 12- and 24-month time points. Finally, the fifth group (chronic high depressive symptoms), which comprised 3.9% of the mothers, presented a high EPDS scores of at least 17 in the antenatal period, decreasing to 15 at 3 months, increasing again to 19 at 12 months, and again decreasing to 17 at 24 months after delivery (Figure).

**Figure:**
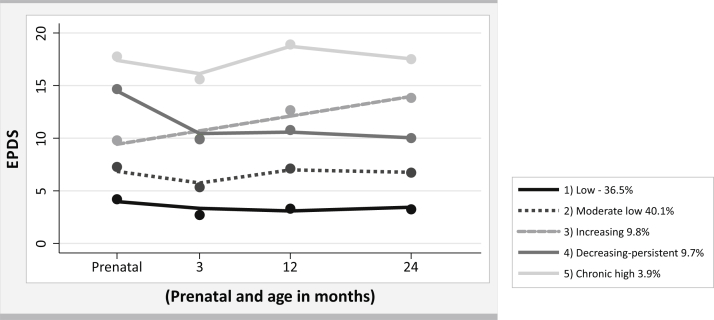
Trajectories of maternal depressive symptoms prenatal and child's age, Pelotas-RS birth cohort 2015

Table V shows the risk of hospitalization in children across the whole period (3, 12, and 24 months) by trajectory groups of maternal depressive symptoms. Compared with children of mothers who were in the group low depressive symptoms, the risk of being hospitalized among children of mothers in the group increasing depressive symptoms was 1.50 (95% CI, 1.17-1.92); for children of mothers who were in the decreasing but persistent symptoms group, the risk of being hospitalized was 1.80 (95% CI, 1.42-2.29); and for the child of a mother in the high chronic depressive symptoms group the risk of being hospitalized was 1.96 (95% CI, 1.46-2.62). We observed an increasing trend in the risk of hospitalization for children in accordance with the severity of trajectories of maternal depressive symptoms at any age in which the children were hospitalized.

**Table V:** Child's hospitalization (3, 12, and 24 months) in accord with trajectories of maternal depressive symptoms from antenatal up 24 months old children, 2015 Pelotas Birth cohort, Brazil

Maternal depressive symptoms (n = 3040)	Crude	Adjusted
Trajectory group	<.001	<.001
Low	1.00	1.00
Moderate-low	1.55 (1.30 1.85)	1.39 (1.16-1.67)
Increasing	1.72 (1.35-2.39)	1.50 (1.17-1.92)
Decreasing	2.35 (1.90-2.90)	1.80 (1.42-2.29)
High chronic	2.74 (2.11-3.55)	1.96 (1.46-2.62)

Adjusted for maternal age, skin color, schooling, income, marital status, depression before pregnancy, planned pregnancy, smoking and alcohol during pregnancy, parity, threat of miscarriage, preeclampsia/eclampsia, and heart disease.

## Discussion

More than one-quarter of the mothers in this Brazilian cohort experienced persistent depressive symptoms from the antenatal period up to 2 years postpartum. The proportion of the children hospitalized between 3 and 12 months of age was higher than between birth and 3 months or 12 and 24 months of age. Moderate antenatal depressive symptoms were not associated with hospitalization at any age at which the child was studied. However, for maternal depression scores classified as significant and severe, we observed a significant association with child's hospitalization. Symptoms of significant and severe antenatal depression were associated with hospitalization in children at 24 months of age, a risk that seemed to be higher when compared with hospitalization at 3 months of age. This study shows that the association between antenatal maternal depressive symptoms and child hospitalization persists and gets stronger for a considerable period after delivery.

To understand this association, it is important to highlight the implications of antenatal and postnatal depression for the women like premature birth, inadequate weight gain, underuse of prenatal care, arterial hypertension and preeclampsia, late onset of breastfeeding, and affects the expression of the placental gene SLC6A4.^10^^,^^38^, ^39^, ^40^, ^41^ These implications and others difficulties like increased risk of social isolation owing to lack of energy, fatigue, and feelings of incompetence, worthlessness, and helplessness and less attachment to the child make it possible that depressed mothers might have struggled in their caring role, and been less responsive to their child's needs, explaining the increased risk for child hospitalization.^15^^,^^42^, ^43^, ^44^, ^45^, ^46^ It is known that children of depressed mothers are more likely to have an inadequate feeding, which results in an increase in the difficulty of breastfeeding, a decrease in the level of self-efficacy of breastfeeding and a reduction in the duration of breastfeeding for the first 6 months of life, and insufficiency nutritional later, besides they are more at risk of having growth retardation, low weight and somatic development delay, diarrhea episode, persistent vomiting, fever, and cough.^11^^,^^47^, ^48^, ^49^ It is also important to note that children in this age group are potentially vulnerable to other important risk factors for diseases, including environmental and social factors, which can increase the risk of hospitalization during a critical period of development of the immune system.^27^^,^^50^ Given that maternal depression may be associated with such risk factors because of common causes, this study cannot establish the causality of the association between maternal depression and child hospitalization. In addition, there are a number of other possible mechanisms that could explain the results, including marital conflict, which might affect the mental health of mothers and, consequently the child's health; contact with the outside world beyond the family environment, such as in day care and where pathogens are easily exchanged; incomplete immunization; and factors common to both maternal depression and child ill-health, such as socioeconomic disadvantages.^1^^,^^5^^,^^51^, ^52^, ^53^

Other studies examining the relationship between antenatal maternal depression symptoms and hospitalization in children under 1 year of age showed that antenatal depressive symptoms are associated with an increase in the risk of admission in a neonatal intensive care unit.^23^^,^^54^^,^^55^ Yet, these studies show the effect of antenatal depression only in the early days of children's lives.

Postnatal depression, studied more frequently than antenatal depression, is considered an important risk factor in many aspects of a children's lives, including their physical, emotional, and psychomotor development.^11^^,^^26^^,^^48^^,^^56^^,^^57^ We find that significant and severe postnatal depressive symptoms at 3 and 12 months are associated with child hospitalization at 12 and 24 months of age, independent of antenatal depressive symptoms. These findings suggest that, among the risk factors for child disease, antenatal and postnatal maternal depressive symptoms are vital to monitor to ensure the health of children.

Our findings are consistent with several studies that have shown that postnatal depressive symptoms increase the risk of hospitalization in children.^20^, ^21^, ^22^ The association found in the Farr et al study indicated reverse causality, because 70% of mothers were diagnosed with depression after the child's hospitalization, thus it was unclear whether maternal depression lead to hospitalization in the child or if it was the opposite.^21^ Minkovitz et al did not find a statistically significant association between maternal depressive symptoms at 2-4 months and 30-33 months and hospitalization in children from birth to 1 year of age; regardless, the data on maternal depressive symptoms were collected after the hospitalization in children.^22^ Unlike the timetable problems of depression symptoms seen in these studies, our study is different because we collected maternal depressive symptoms before the child's hospitalization.

We documented that certain trajectories of maternal depressive symptoms from antenatal to 24 months postpartum are associated with child hospitalization. The trajectory of persistent depressive symptoms represented an increased risk of hospitalization for children, with the trajectory of the group determining the degree of risk. Children of mothers in the high chronic trajectory group seem most vulnerable. Previous studies of maternal trajectory depressive symptoms have examined different outcomes, finding higher levels of internalizing and externalizing problems among children whose mothers showed chronic high levels of depressive symptoms, and these findings underline the need to ensure screening processes for maternal depression in both antenatal and late postpartum periods and put in place appropriate interventions.^58^^,^^59^

A key strength of this study is the examination of maternal depressive symptoms began antenatally and continued until 24 months postnatal, (analyzed individually and as trajectories), and the examination of child hospitalization at multiple time points. In contrast with some studies, maternal depressive symptoms were measured before the child outcomes in this study, to decrease the risk of reverse causality bias. This study also examined risk for children according to different degrees of maternal depressive symptoms, as well as categorizing by depression trajectory groups. With respect to the limitations, only 74% of mothers participating in the Pelotas 2015 Birth Cohort were interviewed during the antenatal period, and only those participants were included in the trajectory analysis. The mothers not included have worse sociodemographic and health conditions, characteristics often associated with symptoms of depression.^13^^,^^60^ This factor may have led to an underestimation of the proportion of mothers with antenatal depressive symptoms and of the risk of children's hospitalization. Data on prenatal and postnatal paternal smoking, other psychiatric disorders, and the treatment of maternal depression were not available; maternal report of child hospitalization might be a limitation but, this reminder was short because the question was repeated at each follow-up. Information about maternal body mass index and postnatal maternal smoking was not included in these analyses. There might be residual confounding in the association because of possible confounders that are not measured by this cohort study.

Maternal depressive symptoms are a risk factor for hospitalization in children until 2 years of age, and this risk increases as the severity of maternal depressive symptoms increases. The State of Rio Grande do Sul is the most prevalent for depression compared with all of the Brazilian states, making these findings relevant for public health and suggesting that targeting risk for depression among mothers could decrease hospitalizations in children.^61^ Quality antenatal and postnatal care are among the best ways to attain this goal, because they can assist in identifying women at risk of developing depressive symptoms in the prenatal and postnatal periods and decrease risk factors for mothers and for morbidity/hospitalization in children.^62^, ^63^, ^64^, ^65^, ^66^, ^67^, ^68^ We recommend a focus on the prevention of women's health in general and maternal and child health in particular and that routine screening for maternal depression be integrated into antenatal and postnatal care.^69^ Further research is needed to understand the association between antenatal and postnatal maternal depressive symptoms and child hospitalization and to test if this relationship is causal.

## Appendix

Data Profile

STROBE checklist
